# Changes in Sexual Desire in Women and Their Partners during Pregnancy

**DOI:** 10.3390/jcm9020526

**Published:** 2020-02-14

**Authors:** Francisco Javier Fernández-Carrasco, Luciano Rodríguez-Díaz, Urbano González-Mey, Juana María Vázquez-Lara, Juan Gómez-Salgado, Tesifón Parrón-Carreño

**Affiliations:** 1 Department of Gynaecology and Obstetrics, Hospiten Group, Hospiten Estepona, 29680 Estepona, Málaga, Spain; franfernanca@hotmail.com; 2 Department of Gynaecology and Obstetrics, Ceuta University Hospital, Midwifery Teaching Unit of Ceuta, Spain, University of Granada, 51003 Ceuta, Spain; lucianin000@gmail.com (L.R.-D.); juani.vazquez@andaluciajunta.es (J.M.V.-L.); 3 Department of Surgery, Punta de Europa Hospital, 11207 Algeciras, Cádiz, Spain; dregenborg@hotmail.com; 4 Department of Sociology, Social Work, and Public Health, University of Huelva, 21007 Huelva, Spain; 5 Safety and Health Post-graduate Programme, Espíritu Santo University, Samborondón 092301, Guayaquil, Ecuador; 6 School of Health Sciences, University of Almeria, 04120 Almeria, Spain; tesifon.parron@juntadeandalucia.es; 7 Territorial Delegation of Equality, Health and Social Policies, Health Delegation of Almeria, Government of Andalusia, 04003 Almeria, Spain

**Keywords:** pregnancy, lifestyle, sexual behavior

## Abstract

When studying sexual desire during pregnancy, most research focuses on the pregnant woman’s sexual desire and almost never takes into account her sexual partner. The novelty of this study is that sexual desire during pregnancy is studied from the point of view of the pregnant woman and from that of her male partner. The goal of this study is to see how sexual desire behaves during pregnancy in both partners. For this, a descriptive, longitudinal, and multistage study was designed. Methodologically, in the first stage, the different study variables were described through a single-variate analysis. In the second stage, one variable was related to others by means of a bivariate analysis. Finally, in the third stage, a multivariate analysis was done, composed of binary logistic regression models and latent growth curves. The results confirm that pregnancy influences the sexual desire of both partners, and that sexual desire behaves differently in women than in men during pregnancy. Men have higher levels of sexual desire throughout pregnancy as compared to women. The first trimester of pregnancy is the period when women have less sexual desire.

## 1. Introduction

The main authors who work on sexual desire established the difficulty of clearly defining erotic desire [1,2]. Sexual desire has a clear biophysiological origin, with a fairly profiled anatomical–neuro-endocrine structure [3,4]. Its function, from an etiological point of view, focuses on the survival of the species through reproduction, i.e., the transmission of the genetic endowment purified by natural selection [5]. Despite the general interest and popular use of nomenclature, scientific knowledge on sexual desire is relatively limited. A consensual definition of sexual desire is yet to be adopted.

In 1966, Masters and Johnson, in their work on human sexual response, developed the first scientific study on the phenomena observed in the organism in response to sexual stimulus and, in it, they distinguished four successive stages, both in the male sexual response and in the female sexual response: excitement, plateau, orgasm, and resolution [6]; however, at no time did they refer to desire. It was Lief who, in 1977, pointed to sexual desire as a different dimension from arousal and orgasm [7] and, later in 1979, Kaplan introduced sexual desire into his three-phase model of sexual response formed by the phases of desire, arousal, and orgasm [8]. In the first stage, desire is the impulse that leads the individual to seek a sexual experience. In the arousal stage, among many other phenomena, the erection of the penis occurs in men and vaginal lubrication occurs in women. Finally, in the third stage, orgasm occurs, during which muscle contractions take place and a feeling of pleasure, concentrated in the genital region and spreading throughout the body, occurs both in men and women [9].

Pregnancy brings about a multitude of physical and psychological changes in women, and psychological changes in men. A slight decrease in sexual interest was reported during the first trimester of pregnancy, followed by a variable pattern of conduct in the second trimester, and a marked decrease in the final period of pregnancy [10,11,12,13,14,15], where even levels defined as sexual dysfunction are reached. In the first three months of pregnancy, an adaptation stage begins, to assume the changes that may occur during the gestation period and the role of parents. Hormonal changes cause a state of emotional lability; thus, the woman may demand more attention and affection on behalf of her partner. It should be noted that many women may require a greater effort to maintain the usual sexual frequency as a result of the onset of nausea, vomiting, repugnance to food and odors, asthenia, and other typical discomforts of this stage such as hypersalivation, headaches, drowsiness, and hypersomnia [16,17]. In the second trimester, the changes observed in the previous stage persist, although many women express that sexual desire increases, with growing interest in coital and manipulative activity. This is related to the fact that fears generally decrease in this stage as compared to the other two, and because they feel greater well-being since physical discomfort is alleviated or disappears [18,19]. In the third trimester, a significant decrease in sexual desire was reported by most women [20]. This is usually due to physical demands given the volume of the maternal abdomen and the feeling of heaviness, the cessation of sexual activity in some cases indicated by the doctor, or psychological issues such as a distorted view of her own body, even considering herself as having little or no attractiveness for the couple due to body changes [13,21].

In the literature, the term sexual desire is understood in two different ways; the first one conceptualizes sexual desire as the impulse to engage in sexual behavior in general [22], while the second one is described as the obligation to engage in sexual intercourse with a particular person [23]. In this study, a differentiation between the two types of sexual desire was attempted. Solitary desire refers to interest in sexual activities that do not involve a partner or may involve abstaining from getting intimate with others. On the contrary, the interest or desire to engage in sexual behavior with another person could serve a different purpose; that is, dyadic desire could also cover the need to intimate with the other [24].

In the scientific literature, many authors developed different techniques to assess sexual desire. Wilson et al. [25], with their sex fantasy questionnaire, measured desire through sexual fantasies, considering that people with more sexual fantasies showed more desire; Masters and Johnson [19], with their inhibited sexual desire test, and the inhibited sexual desire evaluation by Farré and Lasheras [26] aimed at assessing lack of desire rather than desire in itself; likewise, Beck et al.’s sexual desire questionnaire was widely used to assess depression, stating that a depressive person shows little sexual desire [27]. Only Spector et al., with their sexual desire inventory, were the first to differentiate dyadic desire from solitary desire [28].

It is important to assess sexual desire in couples as low or absent sexual desire brings about challenges, not only during the pregnancy period, but Sprecher extends it to romantic relationships in general. Lack of desire can lessen sexual initiation or receptivity, leading to less frequent sexual intercourse and, in turn, threatening the romantic bond by depriving couples of the many benefits of sex, such as intimacy, pleasure, and emotion [29]. The consequences derived from having low sexual desire may imply painful sexual intercourse for women and even impossible sexual intercourse for men, as it limits genital arousal. All this can lead to couple conflicts, infidelity, or breakdown [30].

The goal of this study is to identify changes in sexual desire in both men and women during pregnancy.

## 2. Experimental Section

A descriptive longitudinal study was developed. The studied population consisted of pregnant women and their male partners who visited different hospitals in southern Spain for their first prenatal care visit from January 2017 to December 2018. A sample size was estimated, enough to determine differences of up to nine units between the matched means, with a standard deviation (SD) of the maximum difference of 30, a significance level of 0.05, bilateral contrast, and a power of 0.80. A total of 147 participants were initially recruited, and 39 were excluded because their partners did not wish to participate in the study or because they did not complete the study for any reason. Finally, 108 couples were included in the study, 108 women and 108 men (*n* = 216). This sample was randomly chosen among all women who met the inclusion criteria and whose partners also agreed to participate in the study. The criteria were as described below.

Inclusion Criteria:
  - Pregnant women recruited from the first pregnancy consultation.
  - The male partners of the women attending the consultation.
  - Both Spanish.
Exclusion Criteria:
  - Pregnant women who were not recruited since the beginning of pregnancy.
  - Women who developed some type of pathology during pregnancy.
  - Women whose partner refused to participate in the study.

As a psychometric instrument, the Spector et al. [28] sexual desire inventory was chosen. This scale was upgraded and validated in Spain by Ortega, Sierra, and Zubeidat, showing a high degree of internal cohesion between the items and the reliability of the scale [31]. It includes two domains: dyadic sexual desire and solitary sexual desire. Cronbach’s alpha for dyadic sexual desire was 0.87, and that for solitary desire was 0.88. Although the Spector sexual desire inventory test [28], validated by Ortega, Sierra, and Zubeidat, is a fairly reliable tool as the Cronbach’s alpha was very high [31], in order to confirm that the reliability of this test remained good for our sample, the reliability analysis was performed at the different stages of pregnancy. Through Cronbach’s alpha, it can be stated that this is also a fairly reliable tool for our population. Results were 0.84 at the start of pregnancy, 0.84 in the first trimester, 0.80 in the second trimester, and 0.82 in the third trimester of gestation.

The participants were surveyed four times throughout pregnancy. The first one was in the initial pregnancy consultation, before the ninth week of pregnancy (referring to the pre-pregnancy situation); the second survey took place at week 12 of gestation (first trimester of pregnancy); the third one took place between week 20 and 24 of gestation (second trimester); the fourth and last survey was done between the 32nd week and final week of gestation (third trimester of pregnancy).

During the first visit, participants were informed of the purpose and development of the study. An informed written consent form was signed by both partners prior to participation. The confidentiality of the data was ensured as the information was recorded anonymously. The study received the consent of the directors and was approved by the Research Ethics Committee of the Hospiten Estepona Health Center (Code: PI 01/17).

The variables under study were age, marital status, occupation, level of studies, number of children, and Spector sexual desire inventory score. Statistical analyses were performed using the SPSS software version 23 (Windows, Chicago, IL, USA). Frequencies and percentages were determined for the qualitative variables. Means, maximums and minimums, and standard deviations (SD) were determined for the quantitative variables. The Pearson’s correlation coefficient, Mann–Whitney U test, and Kruskal–Wallis test were used for the bivariate analysis. A *p*-value <0.05 was considered significant. All the study variables were analyzed, relating each of them to the others so as to find associations with statistically significant differences. For the multivariate analysis, binary logistic regression models were performed. For the global behavior analysis, latent growth curves with four mixed-effect linear regression models (ME-LRM) were used for each dependent variable (questionnaire domain scores), considering the random effect of individuals.

This was developed through model I (null), model II (adjusted by the time variable), model III (adjusted by the sex variable), and model IV (adjusted by time and sex (only for those dependent variables whose independent variables were statistically significant in models II and III)).

Model I

Each dependent variable (questionnaire domain scores) was considered and no independent variable was taken into account. The “id” term is specified in the model to consider the random effect of each individual.

Model II

Each dependent variable (questionnaire domain scores) was considered, and time was taken as an independent variable. The “id” term is specified in the model to consider the random effect of each individual.

Model III

Each dependent variable (questionnaire domain scores) was considered, and gender was taken as an independent variable. The “id” term is specified in the model to consider the random effect of each individual.

Model IV

The dependent variables (solitary desire and dyadic desire) were considered because both sex and time were statistically significant or gave signs of statistical significance.

## 3. Results

### 3.1. Descriptive Analysis

The mean age of women was slightly lower, aged 32.72 years ± 4.20, as compared to that of men, who were 33.85 ± 5.46 years. As for the test’s solitary desire domains, the mean value was 14.19 ± 8.43. With dyadic desire, the mean was 50.78, with a standard deviation of 7.40 (Table 1).

As for marital status, most of the participants were married, representing more than 66% of the total sample. These figures were followed by singles, representing almost 28% of participants, and the lowest number was found in those who were divorced, just over 5%. Cases where, although participants could be engaged in a romantic relationship, marriage was not formalized, were considered singles. Those couples where at least one of the members was married to another person, then divorced, before forming another relationship with their current partner without formalizing the marriage, were considered divorced. Based on the level of studies, it was found that the highest percentage of the sample completed university or postgraduate studies (43.1%), followed by those with high school or vocational training (34.7%), and the lowest percentage, 22.2%, represented those who had primary or no education. In terms of the number of children, more than half of the sample (54.2%) did not yet have any children, 33.3% had one child, 11.1% had two children, and 1.4% had three children.

### 3.2. Bivariate Analysis

In the bivariate analysis, statistically significant differences were found between the mean age of women as compared to men, being slightly lower in women (Table 2).

Statistically significant differences were also found in terms of the level of education (*p* < 0.001) with respect to the participants’ sex (Table 3).

Statistically significant differences were also found regarding the participants’ occupation (*p* < 0.001) according to the participants’ sex (Table 4).

As for the test domain score regarding sex comparison, statistically significant differences were found in all measures of both solitary and dyadic desire. In solitary and dyadic sexual desire, men obtained significantly higher scores than women during all trimesters of pregnancy (Table 5).

When comparing the test scores based on the level of studies, statistically significant differences were only found in the dyadic sexual desire domain. A higher dyadic desire was linked to a lower level of studies, and vice versa (Table 6).

When performing the Dunn–Bonferroni post hoc test for dyadic desire with level of studies, statistically significant differences were found between *university/post-graduate* and *high school/vocational training,* and between *university/post-graduate* and *no studies/primary studies,* with *p*-values of 0.048 and 0.024, respectively. No differences were found between *high school/vocational training* and *no studies/primary studies*, with *p* = 0.61.

Dyadic and solitary desires were positively and significantly related to each other. The number of children positively and significantly correlated (Pearson’s correlation) with solitary desire, but did so in a negative and significant way with dyadic desire (Table 7).

**Figure 1:**
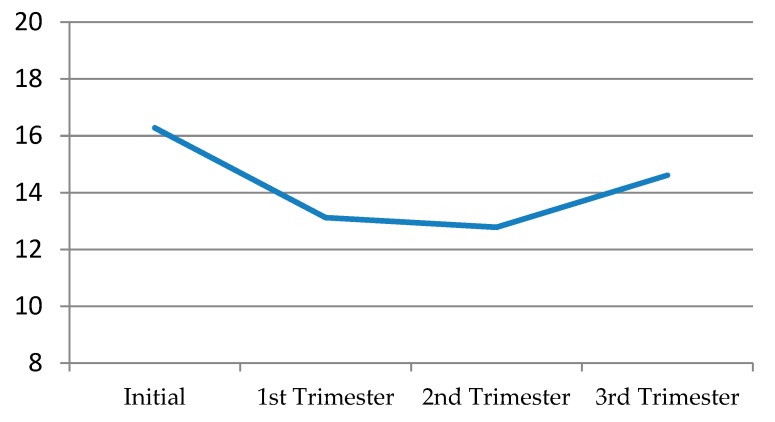
Solitary desire throughout the different pregnancy trimesters.

Solitary desire, in general terms, decreased quite a bit in the first trimester, continued to decline albeit slightly in the second trimester, and then recovered a little in the third, without ever reaching its initial levels (Figure 1) (Table 8).

**Figure 2:**
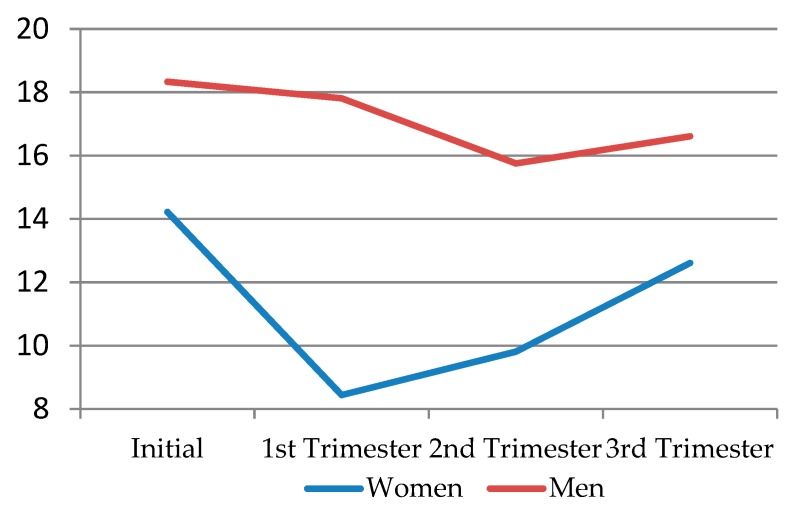
Solitary desire regarding sex in each pregnancy trimester.

When comparing solitary desire according to sex, men’s levels of solitary desire were higher throughout pregnancy than women’s. In the first trimester, solitary desire decreased in both sexes, but this was much more noticeable in women. In the second trimester, it somewhat recovered in women, while males, on the other hand, suffered a small drop in solitary desire levels. In the third trimester, the levels of solitary sexual desire increased in both sexes, but these were always below the initial levels (Figure 2) (Table 8).

When performing the Dunn–Bonferroni post hoc test for solitary desire by trimesters and sex, differences were found between all couples for total solitaire desire, with *p* < 0.002. When selecting only women, differences were also found between all couples for total solitary desire, with *p* < 0.018. Regarding only men, differences were also found between all couples for total solitaire desire, with *p* < 0.001, except for initial solitary desire and the first trimester (*p* = 1).

**Figure 3:**
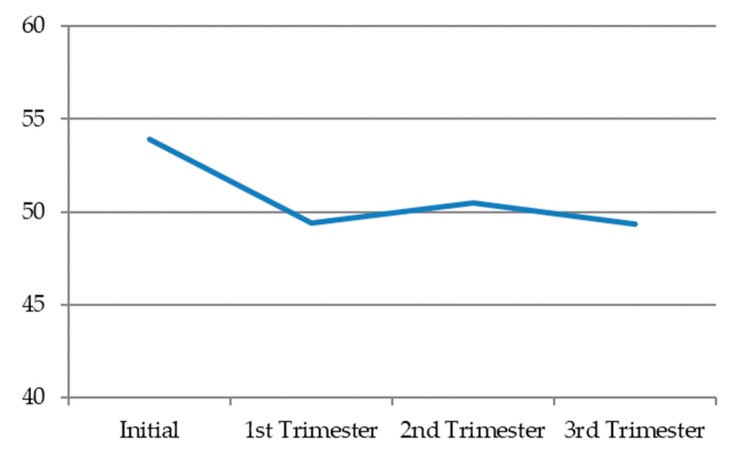
Dyadic desire in each pregnancy trimester.

Dyadic desire, in general terms, decreased during pregnancy. In the first trimester, it reached the lowest levels. Then, in the second trimester, it tended to very slightly recover, before decreasing a little further in the third trimester (Figure 3) (Table 9).

**Figure 4:**
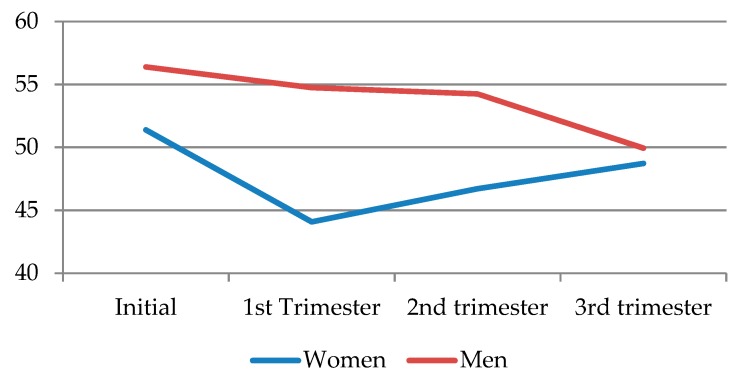
Dyadic desire according to sex for each pregnancy trimester.

When comparing dyadic desire according to sex, it can be seen that, similar to solitary desire, men showed higher levels, but these decreased throughout the pregnancy, resulting in the most noticeable decline toward the third trimester. As for women, dyadic desire markedly declined in the first trimester, before recovering a little toward the second and third trimesters, albeit never reaching the initial levels (Figure 4) (Table 9).

When performing the Dunn–Bonferroni post hoc test for paired data for dyadic desire by trimesters and sex, differences were found between all couples for total solitary desire, with *p* < 0.02, except for couples in the first and third trimesters (*p* = 0.50). When selecting only women, differences were also found between all couples for total solitary desire, with *p* < 0.007. When selecting only men, differences were also found between all couples for total solitary desire, with *p* < 0.001, except when comparing dyadic desire in the first trimester with that in the second (*p* = 0.93).

### 3.3. Multivariate Analysis

*Solitary desire*: Solitary desire was included in the binary logistic regression analysis model as a dependent variable. As independent variables, sex, age, marital status, level of studies, occupation, and children were included. Despite the decline and considering solitary desire throughout gestation, men held six times higher odds than women, divorced participants held 1.23 times higher odds than married participants, and single participants held 4.5 times lower odds than married participants (Table 10).

To demonstrate the adequacy of the model, the Hosmer and Lemeshow goodness-of-fit test was performed (0.767), and, to assess the variance of the dependent variable explained by the model, the Nagelkerke’s *R*-squared model was used (0.414).

Dyadic desire: Dyadic desire was included in the binary logistic regression analysis as a dependent variable and, as independent variables, sex, age, marital status, level of studies, occupation, and number of children were included (Table 11). Despite the decline and considering dyadic desire throughout pregnancy, men held 4.17 times higher scores than women, divorced men held 1.94 times higher scores than married men, and single men held 2.4 times higher scores than married men.

The Hosmer and Lemeshow goodness-of-fit test (0.15) was performed, and, to assess the variance of the dependent variable explained by the model, the Nagelkerke’s *R*-squared model was used (0.18).

Latent growth curves with four mixed-effect linear regression models (ME-LRM) (Table 12).

Model II

An increase in time significantly decreased solitary and dyadic desire by −0.53 and −1.26, respectively (*p* < 0.001).

Model III

Being a man significantly increased solitary and dyadic desire by 5.85 and 6.10, respectively (*p* < 0.001).

Model IV

An increase in time decreased solitary desire by −0.53, keeping the sex variable constant (*p* < 0.001). Being a man increased solitary desire by 5.85, while maintaining the time variable constant (*p <* 0.001).

An increase in time decreased dyadic desire by −1.26 while maintaining the variable sex constant (*p* < 0.001). Being a man increased dyadic desire by 6.10, while maintaining the variable time constant (*p <* 0.001).

## 4. Discussion

Several recent studies claimed that sexual desire in women decreases in the first trimester of pregnancy, remains the same in the second, and further decreases in the third [32,33,34]. These results are attributed to the fact that most studies obtained their data at an isolated stage, i.e., they surveyed a population of pregnant women at just a specific moment of the process, thus obtaining data on women’s sexual desire regarding only one trimester [35]. In this study, the same sample was assessed at four different periods, corresponding to the start of pregnancy and each of the trimesters. This way, it was possible to identify a clear evolution throughout pregnancy.

There were very few studies that focused on male sexual desire during pregnancy. With this study, it can be stated that pregnancy influences the sexual desire of both men and women. However, several studies claimed that the desire in pregnant couples remains the same in all three trimesters of pregnancy [32,36]. These results may be due to the chosen methodology, which was more focused on studying women than their couples. Other studies showed that men’s perception of desire is greater than women’s, and it is maintained with high values until the end of the second trimester, at which point the decline in desire becomes apparent [33,37].

According to Panea et al., women in the third trimester of their pregnancy set aside their sexual appetite and that of their partners to focus on the well-being of their newborn baby. Thus, the role of the mother within the couple was highlighted. Difficult pregnancies led to greater decreases in sexual life [38]. In the present research, these conditions were not met, as it was in the first trimester that sexual desire decreased the most, in terms of both solitary and dyadic desire, in both men and women. Then, in the second and third trimesters, they recovered a little, although never reaching the initial values.

The results of this research show that men express higher levels of desire than women throughout pregnancy. These conclusions may be due to the fact that women, unlike men, tend to exhibit less concordance between their physiological and subjective sexual arousal [39].

The outcomes found in the present study show that dyadic sexual desire decreases in men as pregnancy advances. This may be due to the fact that some may see their partner as less attractive due to the changes that occur in women’s bodies, such as the increase in the size of the abdomen, the fact that the genitals swell at the end of pregnancy, the vagina turns a bluish color due to hyperemia, the breast’s areolas turn dark, a black line appears going from the navel to the pubis, etc. [40]. Men can also see the fetus as an intruder in the relationship or as a third person, making them feel uncomfortable regarding sexual encounters. In addition, due to the changing roles in the couple, the woman may be regarded as a mother instead as the object of sexual desire she was before. Of course, men may also fear of harming the fetus as a result of sexual encounter [41].

This study also had a significant limitation, which was the decision to consider just age, marital status, occupation, level of education, and number of children as independent variables. In fact, differences in individuals’ sexual desire among the different gestational phases could also be due to other psychological factors that might be present and that were not assessed in this study (e.g., presence of depression, lack of psychological well-being, distress, stress, dysfunctional beliefs, etc.) [42,43,44].

## 5. Conclusions

Sexual desire is altered by pregnancy, decreasing as the pregnancy progresses. Solitary desire decreases considerably more for women than for men during the first trimester of pregnancy. Dyadic desire also drops in both partners during pregnancy; however, unlike women, men have their lowest dyadic desire levels in the third trimester. In contrast, in this same period, their level of solitary desire increases. The period of lower sexual desire for women, both solitary and dyadic, is the first trimester of pregnancy. Sexual desire levels are always higher in men throughout pregnancy.

In this study, we found a number of limitations. The first was that, although there were numerous studies on sexual desire, very few linked this concept to pregnancy. Thus, the literature found was sparse and a little old-fashioned. This fact makes our study a novelty. On the other hand, we found limitations when evaluating the sample. It was collected among patients who visited several hospitals in southern Spain; thus, we cannot extrapolate the results to a more heterogeneous population. It would be interesting, and we leave this door open for future research, if this same study were to be carried out in a much wider population, taking samples from different countries, in order to be able to extrapolate the results.

It would also be interesting to be able to study how sexual desire is affected beyond childbirth, that is, to be able to check how this behavior changes during postpartum and the first years of child-rearing.

## Figures and Tables

**Table 1:** Mean values of the studied quantitative variables.

Variables	*N*	Minimum	Maximum	Mean	SD
Women age	108	24	43	32.72	4.20
Men age	108	21	48	33.85	5.46
Solitary desire	216	0.00	30.50	14.19	8.43
Dyadic desire	216	34.00	67.50	50.78	7.40

**Table 2:** Mean age regarding the participants’ sex.

Sex	*N*	Minimum	Maximum	Mean	SD	*p*-Value *
Women	108	24	43	32.72	4.20	<0.001
Men	108	21	48	33.85	5.46	

* Statistical significance (*p* < 0.001).

**Table 3:** Distribution of the level of studies according to sex.

		No Studies/Primary	High School/Vocational	University/Post-Graduate	Total
Woman	Count	12	36	60	108
	% according to sex	11.1%	33.3%	55.6%	100.0%
Man	Count	36	39	33	108
	% according to sex	33.3%	36.1%	30.6%	100.0%

Chi-squared = 19.95 (*p* < 0.001).

**Table 4:** Occupation distribution according to sex.

		Studies	Works	Household/Family Care	Unemployed	Total
Woman	Count	3	72	12	21	108
	% according to sex	2.8%	66.7%	11.1%	19.4%	100.0%
Man	Count	3	102	0	3	108
	% according to sex	2.8%	94.4%	0.0%	2.8%	100.0%

Chi-squared (likelihood ratio) = 37.02 (*p* < 0.001).

**Table 5:** Comparison by sexes of the test domain scores.

Domains	Sex	*N*	Mean	SD	*p*-Value *
Initial solitary desire	Woman	108	14.22	8.55	0.001
	Man	108	18.33	8.13	
1st trimester solitary desire	Woman	108	8.44	7.59	0.001
	Man	108	17.81	8.59	
2nd trimester solitary desire	Woman	108	9.81	8.10	0.001
	Man	108	15.75	7.84	
3rd trimester solitary desire	Woman	108	12.61	8.77	0.001
	Man	108	16.61	8.63	
Initial dyadic desire	Woman	108	51.39	7.18	0.001
	Man	108	56.39	6.29	
1st trimester dyadic desire	Woman	108	44.08	7.84	0.001
	Man	108	54.75	6.92	
2nd trimester dyadic desire	Woman	108	46.72	8.07	0.001
	Man	108	54.25	7.36	
3rd trimester dyadic desire	Woman	108	48.72	7.06	0.001
	Man	108	49.94	8.60	

* Mann–Whitney’s U.

**Table 6:** Comparison by level of studies of the test domain scores.

Domains	Studies	*N*	Mean	SD	*p* Value *
Solitary desire	No studies or primary studies	48	15.67	7.61	0.16
	High school or vocational training	75	14.93	8.56	
	University or post-graduate	93	12.84	8.62	
Dyadic desire	No studies or primary studies	48	52.26	7.88	0.04
	High school or vocational training	75	51.64	7.25	
	University or post-graduate	93	49.32	6.84	

* Kruskal–Wallis.

**Table 7:** Correlation between both types of desire and number of children.

		Solitary Desire	Dyadic Desire
No. of children	Significance correlation coefficient	0.135	−0.184
	(bilateral)	0.04	0.007
Solitary desire	Significance correlation coefficient		0.360
	(bilateral)		0.001

**Table 8:** Total solitary desire by sex for each pregnancy trimester.

	Solitary Desire	Mean	SD	*p*-Value *
Total	Initial	16.28	8.57	0.001
	1st trimester	13.13	9.35	
	2nd trimester	12.78	8.49	
	3rd trimester	14.61	8.91	
Women	Initial	14.22	8.55	0.001
	1st trimester	8.44	7.59	
	2nd trimester	9.81	8.10	
	3rd trimester	12.61	8.77	
Men	Initial	18.33	8.13	0.001
	1st trimester	17.81	8.59	
	2nd trimester	15.75	7.84	
	3rd trimester	16.61	8.63	

* Kruskal–Wallis.

**Table 9:** Total dyadic desire by sex for each pregnancy trimester.

	Dyadic Desire	Mean	SD	*p*-Value *
Total	Initial	53.89	7.18	0.001
	1st trimester	49.42	9.11	
	2nd trimester	50.49	8.58	
	3rd trimester	49.33	7.87	
Women	Initial	51.39	7.18	0.001
	1st trimester	44.08	7.84	
	2nd trimester	46.72	8.07	
	3rd trimester	48.72	7.06	
Men	Initial	56.39	6.29	0.001
	1st trimester	54.75	6.92	
	2nd trimester	54.25	7.36	
	3rd trimester	49.94	8.60	

* Kruskal–Wallis.

**Table 10:** Binary logistic regression for solitary desire. CI—confidence interval; OR—odds ratio.

	OR	95% CI for OR		Significance
		Lower	Higher	
**Man**	**6.295**	3.159	12.542	0.0001
Married	Reference			0.001
Single	0.224	0.105	0.475	0.0001
Divorced	1.229	0.325	4.658	0.761

Dependent variable: solitary desire. Independent variables: sex, age, marital status, level of studies, occupation, number of children.

**Table 11:** Binary logistic regression for dyadic desire.

	OR	95% CI for OR		Significance
		Lower	Higher	
**Man**	**4.175**	2.326	7.494	0.000
Married	Reference			0.023
Single	2.474	1.273	4.808	0.008
Divorced	1.942	0.557	6.769	0.297

Dependent variable: dyadic desire. Independent variables: sex, age, marital status, level of studies, occupation, number of children.

**Table 12:** Analysis of mixed models (regression coefficients with standard errors).

**Solitary Desire**	**Model I**	**Model II**	**Model III**	**Model IV**
*Fixed effects*				
Intercept	14.19 (0.57)	15 (0.59)	11.27 (0.76)	12.07 (0.77)
Time		−0.53 * (0.10)		−0.53 * (0.10)
Sex			5.85 * (1.07)	5.85 * (1.07)
*Random effects*				
Variance (intercept)	67.94 (6.82)	68.06 (6.82)	59.37 (5.99)	59.49 (5.99)
Variance (residual)	11.77 (0.65)	11.29 (0.62)	11.77 (0.65)	11.29 (0.62)
**Dyadic Desire**	**Model I**	**Model II**	**Model II**	**Model IV**
*Fixed effects*				
Intercept	50.78 (0.50)	52.67 (0.54)	47.72 (0.64)	49.61 (0.67)
Time		−1.26 * (0.13)		−1.26 * (0.13)
Sex			6.10 * (0.91)	6.10 * (0.91)
*Random effects*				
Variance (intercept)	49.13 (5.25)	49.80 (5.25)	39.82 (4.36)	40.48 (4.35)
Variance (residual)	21.58 (1.19)	18.94 (1.05)	21.58 (1.19)	18.94 (1.05)

* Statistical significance (*p* < 0.001.
